# Mapping Adolescent Suicidal and Non-Suicidal Self-Injurious Behaviours Across Eastern Australia

**DOI:** 10.1007/s10900-025-01523-0

**Published:** 2025-11-19

**Authors:** Ryan Baldwin, Glenn Melvin, Michael McGrath, Ziad Nehme, Harry M. X. Lai, Matthew Green, Dan I. Lubman, Rowan. P. Ogeil

**Affiliations:** 1 Eastern Health Clinical School, Monash University, Box Hill, VIC Australia; 2 Turning Point, Eastern Health, 110 Church Street, Richmond, VIC Australia; 3 Monash Addiction Research Centre, Monash University, Frankston, VIC Australia; 4 SEED Lifespan, School of Psychology, Faculty of Health, Deakin University, Burwood, VIC Australia; 5 Discipline of Psychiatry and Mental Health, School of Clinical Medicine, University of New South Wales, Sydney, Australia; 6 School of Public Health and Preventive Medicine, Monash University, Melbourne, VIC Australia; 7 Ambulance Victoria, Blackburn North, VIC Australia; 8 Department of Paramedicine, Monash University, Frankston, VIC Australia; 9 Health Data Intelligence, New South Wales Ambulance, State Operations Centre, Sydney, NSW Australia; 10 Faculty of Medicine and Health, University of Sydney, Sydney, NSW Australia; 11 Tasmanian Department of Health, Tasmanian State Government, Hobart, Australia

**Keywords:** Adolescents, Self-Harm, Ambulance attendance, Mental health, Suicide

## Abstract

In Australia, adolescent suicidal and non-suicidal self-injury (NSSI) behaviours have been steadily increasing over that past two decades. However, existing research on the geographic distribution of these behaviours in Australia largely focuses on adults, suicides, or uses pre-COVID-19 data. This study aimed to map and describe the geographic regions where ambulance attendances for adolescent suicidal and NSSI behaviours are occurring. Data from the National Ambulance Surveillance System were used to analyse three-year averages and per capita rates of ambulance attendances for adolescents (12–17 years) across local government areas in New South Wales (NSW), Victoria, and Tasmania from 2021 to 2023. The three-year annual average of ambulance attendances for adolescent suicidal behaviour and NSSI were 6,504 in NSW (35.94 per 10,000), 4,017 in Victoria (25.61 per 10,000), and 444 in Tasmania (44.86 per 10,000). Nearly 90% of attendances required transport to hospital, and over 50% occurred outside traditional service hours. Results identified that Major Cities and high socioeconomic status (SES) local government areas (LGAs), were significantly less likely to have higher rates of ambulance attendances for adolescent suicidal and NSSI behaviours. This study highlights substantial variability across three eastern states of Australia where adolescent suicidal and NSSI behaviours are occurring, demonstrating the need for accessible after-hours mental health support, and that regional and lower SES LGA’s are disproportionately impacted. Given the concerning state of adolescent mental health in Australia, this study highlights the need for targeted, data-informed responses to reduce the current reliance on emergency care.

**Supplementary Information:**

The online version contains supplementary material available at 10.1007/s10900-025-01523-0.

Suicide is one of the leading global causes of death for adolescents [1], and in Australia, hospitalisations for suicidal and non-suicidal self-injury (NSSI) have been steadily increasing since 2008 [2]. A major barrier to Australian suicide prevention is the limited availability, both temporally and geographically, of mental health services, particularly in regional and remote areas [3]. Understanding where adolescents experience suicidal and NSSI behaviours is essential for placing mental health services and implementing effective suicide prevention strategies.

Australian geospatial suicide prevention literature has informed mental health service provision by identifying at-risk areas or clusters of suicide [4–7]. However, much of this work focuses on adult populations, is limited to death by suicide, or reports data that predates COVID-19, which influenced adolescent mental health trends [8]. These factors mean that current decision making, and policy evaluation is limited when informing current adolescent suicide prevention. To effectively inform service planning and suicide prevention strategies, it is important to understand where precursor behaviours that result in healthcare utilisation, such as suicidal behaviours, are occurring [9, 10]. Although hospital separation data has been used to identify high-risk areas for suicidal and NSSI behaviours [7], the non-specificity of ICD-10 codes may omit up to 50% of intentional self-injury cases [11]. Geospatial analysis using population surveys has attempted to address this gap [12], but likely underestimate suicidal and NSSI behaviour prevalence, particularly among high-risk cohorts such as those with a history of mental health hospitalisation [13]. The National Ambulance Surveillance System (NASS) [14] offers a unique opportunity to address these gaps by identifying where and when adolescents are seeking emergency care for suicidal behaviour and NSSI.

The NASS is an internationally unique, specialised surveillance system that systematically codes paramedic case-notes to identify all suicidal and NSSI related ambulance attendances. By utilising NASS data on the locations of ambulance attendances for adolescent suicidal and NSSI behaviours, policymakers can be better informed where these acute harms occur and if communities are disproportionally impacted. This information is critical for identifying geographical and temporal service gaps and informing targeted suicide prevention interventions, particularly in regional communities where, uptake and availability of mental health care services is significantly lower compared with metropolitan areas [15–17]. The current study aims to address this knowledge gap by using post-COVID-19 data to map and describe the geographic regions where ambulance attendances for adolescent suicidal and NSSI behaviours are occurring.

## Method

The current study utilises data from the NASS to identify ambulance attendances to adolescents (12–17 years) for suicidal and NSSI behaviours across New South Wales (NSW), Victoria, and Tasmania. Together the estimated adolescent population (12–17 years) of NSW (609,960), Victoria (482,714) and Tasmania (41,043), account for 58% of Australia’s adolescent population (4% of Australia’s total population) [18]. In Australia, ambulance services are managed by each state or territory, offering free or subsidised coverage, ensuring near-universal access. A team of specially trained coders assessed paramedic case notes to determine whether within the last 24 h, the patient had experienced physical or psychological harms related to suicide attempt, suicidal ideation or NSSI. Details underlying the development of the NASS and of coding decisions are explained in further detail elsewhere [14]. Ambulance attendances for adolescent suicidal and NSSI behaviours that occurred between January 1 st 2021 and December 31 st 2023 were utilised in the current study. Ethics approval for NASS is granted through the Eastern Health Human Research Ethics Committee.

### Geospatial and Outcome Information

Using the postcode of the ambulance attendance, attendances were aggregated by local government area (LGA), and attendances with a missing postcode were removed from the study (0.72% of all attendances). Both total number (counts) and per capita rates of ambulance attendances were examined in the current study. To determine per capita rates of ambulance attendances, 2021 census count data for adolescents aged 12–17 years per LGA were obtained from Australian Bureau of Statistics [19], and aggregated LGA counts were divided by LGA population counts to determine ambulance attendances per 10,000 adolescent population. To reduce the influence of anomalous clusters of suicidal and NSSI episodes and to provide a more accurate demonstration of yearly averages and per capita rates, ambulance attendances within each LGA were averaged over the three-year period. A shapefile (digital file of boundaries) of 2021 LGA’s [20] was then used to present the population rates of ambulance attendances for suicidal and NSSI behaviours.

### Geospatial Descriptors

Socioeconomic status of each LGA was approximated using the Index of Relative Socio-economic Advantage and Disadvantage [IRSAD: 21]. The IRSAD is a general measure of socioeconomic advantage and disadvantage, ranking areas from higher disadvantage (1) to lack of disadvantage (10) based on factors including income, education, and employment. Remoteness classification (major city; inner regional; outer regional; remote/very remote), that is the LGA’s relative geographical access to services and amenities, was assigned to each LGA [22].

### Analysis Plan

Three year (2021–2023) average counts and rates of adolescent suicidal and NSSI ambulance attendances were aggregated at the LGA level and visualised using choropleth maps. Each LGA was then classified by remoteness per the Australian Statistical Geography Standard (ABS, 2016), by socioeconomic decile using the IRSAD, collapsed into tertiles (low [1–3] , medium [4–7] and high [8–10]) and grouped into quartiles according to attendance ranking, where the highest quartile represents the LGAs with the highest average count/rate of NSSI ambulance attendances. As average attendance counts and rates were positively skewed, non-parametric Kruskal–Wallis H tests [23] with post hoc Dunn tests for multiple comparisons (with Bonferroni corrections) and evaluation of mean ranks [24] were used to assess differences in attendances across remoteness and IRSAD groups.

## Results

Table 1 describes the total number and per capita rate of ambulance attendances for adolescent suicidal and NSSI behaviours over the three-year study period (2021–2023). Each state appears to have different trends in adolescent ambulance attendances for suicidal and NSSI behaviours, with Victoria decreasing from 2021, NSW decreasing from 2021 to 2022, but increasing from 2022 to 2023, and Tasmania saw consistent increases in attendances since 2021.

**Table 1:** Description of yearly adolescent suicidal and non-suicidal self-injury ambulance attendances

		Yearly suicidal and NSSI ambulance attendance count (rate per 10,000)		
		New South Wales	Victoria	Tasmania
Yearly attendances				
	− 2021	6,942 (39.69)	4,945 (35.64)	335 (30.12)
	− 2022	5,840 (33.39)	3,700 (26.67)	476 (42.80)
	− 2023	6,730 (38.48)	3,405 (24.54)	522 (46.93)
	− 3-Year Average	6,504 (35.94)	4,017 (25.61)	444 (44.86)
Service descriptors				
	- Transport to hospital (%)	95%	91%	86%
	- After Hours Attendances ^a^ (%)	63%	63%	65%

^a^Ambulance dispatches from 17:01–07:59. NSSI = non-suicidal self-injury

### Average Yearly Counts of Adolescent Attendances for Suicidal and NSSI Behaviours

**Fig. 1:**
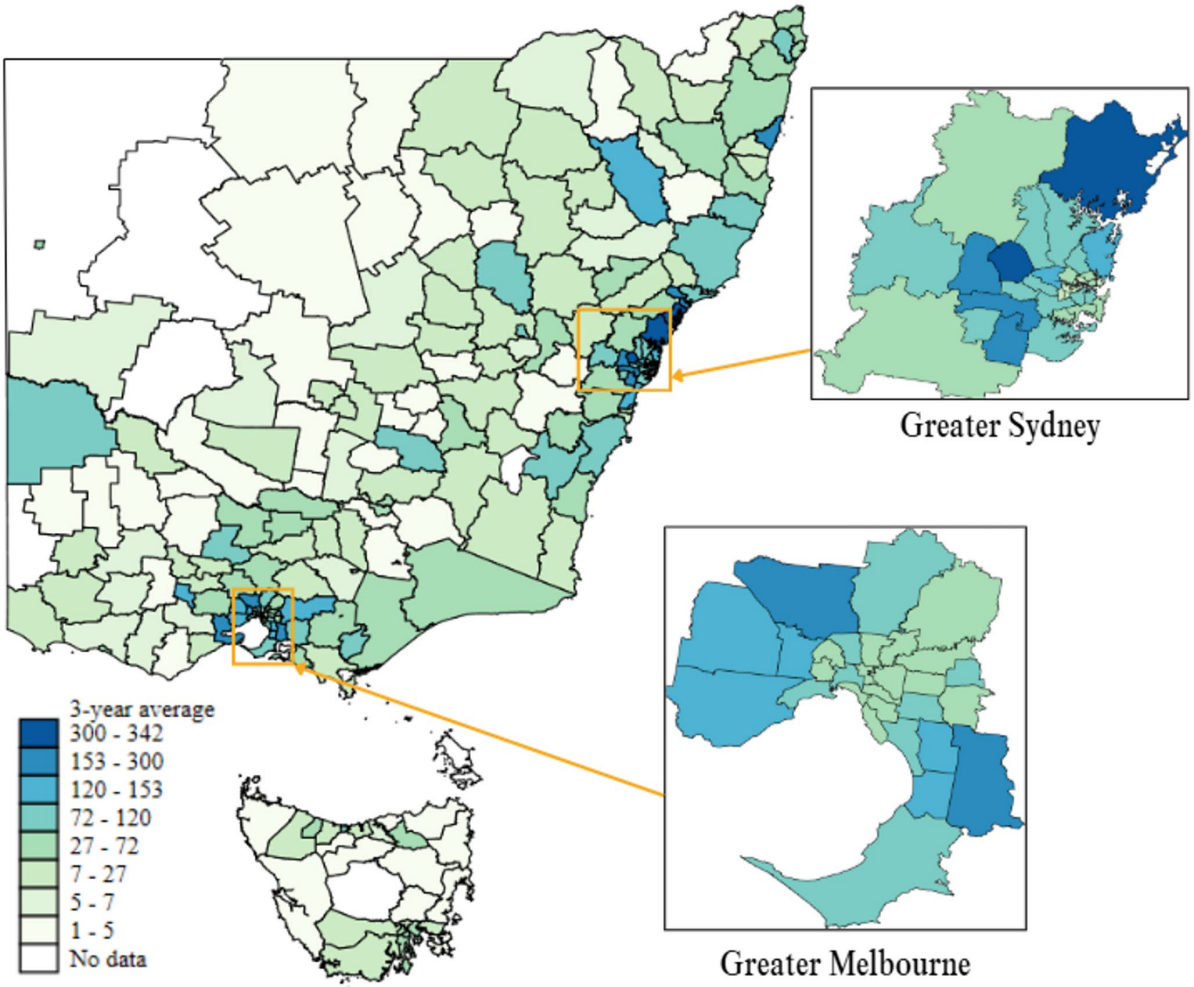
Local government area level choropleth map of the three-year average counts of adolescent suicidal and non-suicidal self-injury ambulance attendances. *Note: *Hotspot scaling was determined following the distribution of the three-year average rate (i.e., 1 st group = 1 st percentile, 5th group = 50th percentile, 9th group = 99th percentile)

Figure 1 presents an LGA level choropleth map of the three-year average count of adolescent suicidal and NSSI ambulance attendances. Areas with dark blue represent those LGAs with the highest yearly average of adolescent suicidal and NSSI ambulance attendances, whereas light green represent LGAs with the lowest rates of yearly averages. Metropolitan areas, particularly around greater Melbourne and Sydney, appear to be where a large number of adolescent suicidal and NSSI ambulance attendances occur. Across all the jurisdictions, there was a significant difference in the distribution of ambulance attendance by remoteness category χ^2^(3) = 97.77, *p* <.001 (see Table 2). Dunn post-hoc analysis with Bonferroni adjustments and evaluation of mean ranks indicated that major city LGA’s were more likely to be in highest attendance quartile compared to inner regional (*z* = 6.03, *p* <.001), outer regional (*z* = 9.48, *p* <.001), and remote and very remote LGA’s (*z* = 5.65, *p* <.001). Inner regional LGA’s were more likely to be in higher attendance quartiles compared to outer regional (*z* = 4.36, *p* <.001) and remote and very remote LGA’s (*z* = 3.01, *p* =.008). Across all the jurisdictions, there was a significant difference in the distribution of rates of ambulance attendance by IRSAD ranking χ^2^ (2) = 25.83 *p* <.001. Dunn post-hoc analysis with Bonferroni adjustments and evaluation of mean ranks indicated that LGA’s within the high IRSAD bracket (8–10) were significantly more likely to be within the higher quartiles of attendances compared to low (*z* = −4.96, *p* <.001) and medium (*z* = −3.88, *p* <.001) SES LGA’s. There was no difference between low and medium SES LGA’s in the number of attendances. A full list of LGA ambulance attendance three-year average count can be found in supplementary material Table S1.

**Table 2:** Distribution of local government areas across attendance quartiles, by remoteness and index of relative socio-economic advantage and disadvantage grouping

Category	Q1 (*n*/%)	Q2 (*n*/%)	Q3 (*n*/%)	Q4 (*n*/%)	Total
*Remoteness*					
Major city	1 (1.37%)	5 (6.85%)	26 (35.62%)	41 (56.16%)	73
Inner regional	22 (23.91%)	26 (28.26%)	29 (31.52%)	15 (16.30%)	92
Outer regional	29 (51.79%)	23 (41.07%)	3 (5.36%)	1 (1.79%)	56
Remote/very remote	7 (77.78%)	2 (22.22%)	0 (0.00%)	0 (0.00%)	9
*Socioeconomic status (SES)*					
Low SES^a^	21 (36.84%)	19 (33.33%)	11 (19.30%)	6 (10.53%)	57
Medium SES^a^	30 (32.26%)	25 (26.88%)	15 (16.13%)	23 (24.73%)	93
High SES^a^	8 (10.00%)	12 (15.00%)	32 (40.00%)	28 (35.00%)	80

Q1 = Lowest attendance quartile, Q4 = Highest attendance quartile.

^a^SES based on Index of Relative Socio-economic Advantage and Disadvantage rankings.

### Per Capita Rates of Adolescent Attendances for Suicidal and NSSI Behaviours

**Fig. 2:**
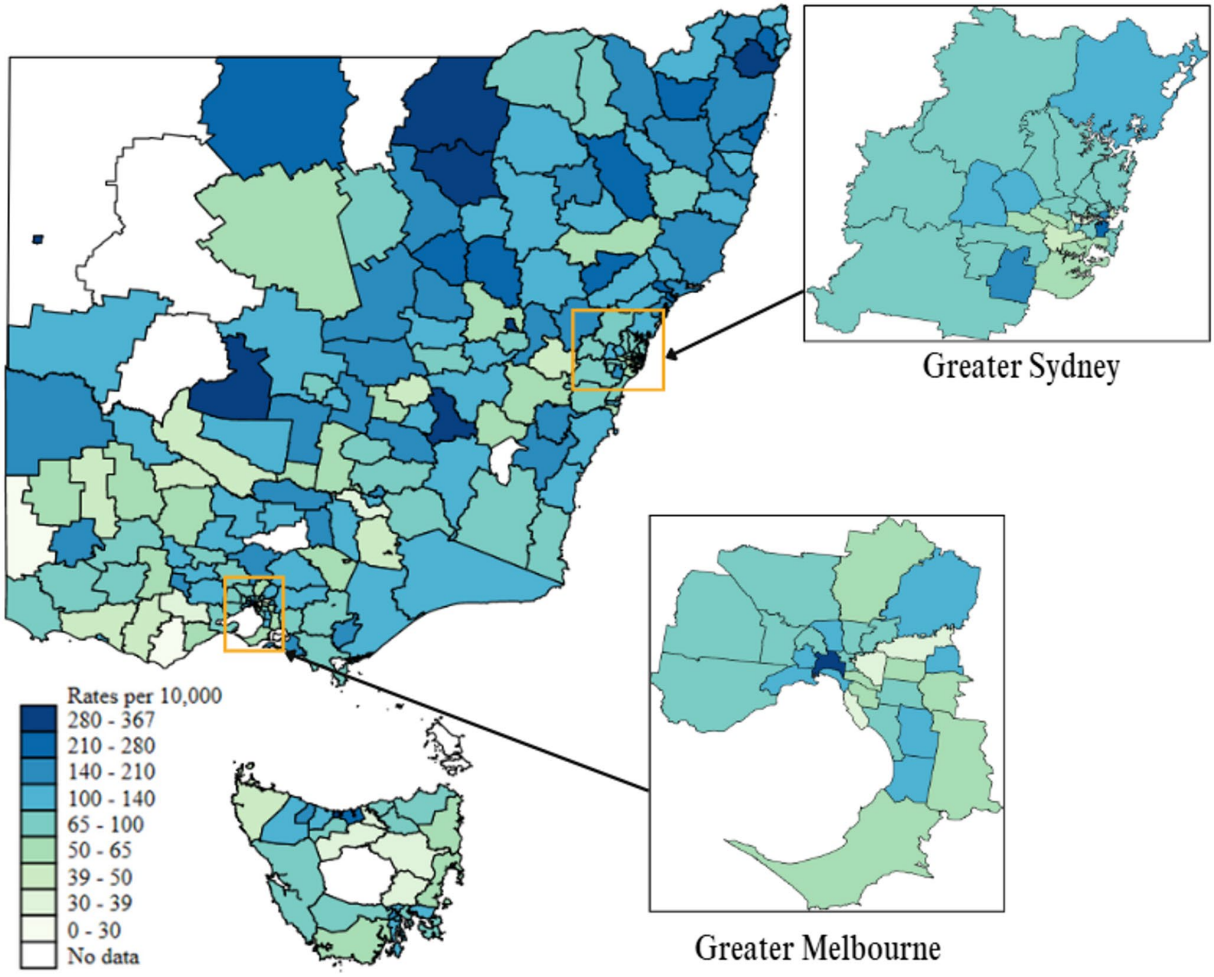
Local government area level choropleth map of the three-year average per capita rates of adolescent suicidal and non-suicidal self-injury ambulance attendances. *Note*: Hotspot scaling was determined following the distribution of the three-year average rate (i.e., 1 st group = 1 st percentile, 5th group = 50th percentile, 9th group = 99th percentile)

Figure 2 presents an LGA level choropleth map of the three-year average per capita rates of adolescent suicidal and NSSI ambulance attendances. Areas with dark blue represent those LGAs with the highest rate of adolescent suicidal and NSSI ambulance attendance, whereas light green represents LGAs with the lowest rates. Inner and outer regional areas, particularly within Western/Far West NSW, appear to have disproportionally higher rates of ambulance use for suicidal and NSSI behaviours compared to metropolitan population centres. Across all the jurisdictions, there was a significant difference in the distribution of rates of ambulance attendance by remoteness category χ^2^(3) = 10.35, *p* =.02 (see Table 3). Dunn post-hoc analysis with Bonferroni adjustments and evaluation of mean ranks indicated that major city LGA’s were less likely to be in higher attendance quartiles compared to inner regional (*z* = −3,08, *p* =.007) and outer regional (*z* = −2.41, *p* =.048) LGA’s. Across all the jurisdictions, there was a significant difference in the distribution of rates of ambulance attendance by IRSAD ranking χ^2^(2) = 24.27, *p* <.001. Dunn post-hoc analysis with Bonferroni adjustments and evaluation of mean ranks indicated that higher SES LGA’s were less likely to be in higher attendance quartiles compared to low (*z* = 4.59, *p* <.001) and medium SES (*z* = 4.14, *p* <.001) LGA’s. A full list of LGA ambulance attendance rates can be found in supplementary material Table S1.

**Table 3:** Distribution of local government areas across attendance quartiles, by remoteness and index of relative socio-economic advantage and disadvantage grouping

Category	Q1 (*n*/%)	Q2 (*n*/%)	Q3 (*n*/%)	Q4 (*n*/%)	Total
*Remoteness*					
Major city	21 (28.77%)	29 (39.73%)	15 (20.55%)	8 (10.96%)	73
Inner regional	22 (23.91%)	14 (15.22%)	28 (30.43%)	28 (30.43%)	92
Outer regional	13 (22.81%)	13 (22.81%)	14 (24.56%)	17 (29.82%)	57
Remote/very remote	2 (22.22%)	2 (22.22%)	1 (11.11%)	4 (44.44%)	9
*Socioeconomic status (SES)*					
Low SES^a^	11 (19.30%)	8 (14.04%)	15 (26.32%)	23 (40.35%)	57
Medium SES^a^	20 (21.28%)	18 (19.15%)	27 (28.72%)	29 (30.85%)	94
High SES^a^	27 (33.75%)	32 (40.00%)	16 (20.00%)	5 (6.25%)	80

Q1 = Lowest attendance quartile, Q4 = Highest attendance quartile.

^a^SES based on Index of Relative Socio-economic Advantage and Disadvantage rankings.

## Discussion

The current study aimed to identify, map and describe the geographic regions where ambulance attendances for adolescent suicidal and NSSI behaviours are occurring across three eastern Australian states. This study highlights that although there is great variability across where ambulance attendances for adolescent suicidal and NSSI behaviours are occurring, regional and remote LGA’s and lower SES LGA’s are disproportionately impacted. This study demonstrates the value and utility of ambulance attendance data in identifying areas of high service demand, which can be used to inform service planning, and evaluate models of care. With nearly all ambulance attendances requiring a transport to the ED, and more than half of all ambulance attendances occurring after traditional work hours, the current paper highlights that adolescent suicidal and NSSI behaviours are not evenly distributed across regions, demonstrating a clear need for targeted resource allocation, and expansion of after-hours mental health support services.

As is to be expected due to population sizes, when examining the average number of attendances by LGA, the highest number of ambulance attendances were observed in major city and inner regional LGA’s. However, when examining per capita rates of suicidal and NSSI behaviours by LGA, we found the opposite, with major cities having the lowest rates of ambulance attendances compared to inner and outer regional LGA’s. Similarly, high SES LGA’s were clustered in higher attendance quartiles compared to low and medium LGA’s, demonstrating that more regional and disadvantaged areas experienced the highest rates of ambulance attendances for adolescent suicidal and NSSI behaviours.

These findings mirror the wider literature that has consistently demonstrated higher rates of suicidal and self-harm behaviours within regional or remote areas [12, 25]. However, the current findings extend this knowledge by highlighting adolescents in these regions are using ambulances to attend to suicidal and self-injury crises at a much higher rate compared to metropolitan regions. It is possible this is due to broader structural inequities in these areas resulting in a disproportionate amount of mental health problems [26]; however, it is also possible that in these regional and remote areas there is a lack of primary or secondary care options [15], resulting in emergency responses becoming the only available or reliable option for care. Access to timely, affordable and well-resourced mental health care in regional and rural communities is consistently identified as a barrier for individuals, particularly adolescents, to obtaining assistance for mental health [3, 27]. Many regional and remote areas community serve extremely large catchment areas spanning hundreds of thousands of square kilometres. This can result in community or inpatient mental health services being located long distances from residential areas, making them particularly difficult to reach in times of crisis. Given these challenges, it is understandable that ambulances are commonly called and adolescents are transported to emergency departments, as other forms of primary or secondary care may be limited or geographically inaccessible.

It is currently very difficult to estimate the number of mental health providers within a given region, as there are numerous public and private specialised and generalised services that offer mental health services to adolescents [28, 29]. To better estimate the relationship between high-risk areas and determination of what services are needed in those regions, a systematic mapping, or creation of mental health directory is needed across Australia [17]. This would assist in reducing the service-related barriers to seeking non-emergency care, and allow for expansion and innovation to both physical and digital health services, such as the expansion of telehealth, in regions where services are currently limited or difficult to access. The expansion of telehealth and telepsychiatry since COVID-19 has improved access to mental health care across Australia and is associated with reduced suicide risk [30, 31]. Early research shows promising outcomes for telehealth use in adolescents experiencing acute self-harm, including symptom reduction and fewer ED transports [32, 33]. However, face-to-face care remains preferred by many young people in regional and remote areas [34]. Alternative models, such as hybrid approaches like the HOPE suicidal behaviour aftercare service [35], may offer a more scalable solution to reducing the use emergency care systems, compared to solely expanding physical infrastructure. Although HOPE is currently adult-focused, youth-specific adaptations are under evaluation [36]. Additionally, brief ED-delivered intervention, such as safety planning combined with digital tools and follow-up messages, also show potential to support adolescent’s and their families post-crisis [37–39].

There were notable state-by-state discrepancies in overall rate trend in ambulance attendances for adolescent suicidal and NSSI behaviours, with Tasmania showing an increase across the study period, Victoria demonstrating a steady decline, and New South Wales experiencing an initial decrease followed by an increase. These differing patterns likely reflect a range of underlying factors, including differences in service availability, mental health system capacity, and state-specific policy or operational changes. In Victoria, for example, rates of adolescent self-harm peaked during the COVID-19 lockdowns [8]. However, subsequent reforms to Ambulance Victoria’s dispatch protocols may have also influenced trends in service use. Specifically, changes to the dispatch grid allowed callers presenting with mental health-related distress to be triaged by a mental health nurse, offering alternative, non-emergency, care pathways for low-acuity presentations. This model evolved from an earlier video consultation triage trial, which reported reduced ambulance dispatches and high patient satisfaction [40]. A similar shift in low-acuity triage protocols in 2016 led to a significant reduction in unnecessary ambulance deployments [41]. Such system-level changes likely contributed to the observed reductions in ambulance attendances in Victoria and may point to opportunities for more targeted and efficient crisis response strategies across other jurisdictions.

### Limitations

The current study used an estimated LGA population to determine rates of attendances per LGA, and, as such, this method is unable to account for areas with high rates of visitors (i.e., metropolitan regions), which may partially explain the higher averages in those areas. Additionally, the current study explored average attendances, not the number of individuals, and as such it is possible that numbers are being skewed by individuals with frequent ambulance attendances, and may not reflect the trends within the entire LGA. This may be particularly pronounced for smaller jurisdictions. Known at-risk groups may be more likely to have multiple lifetime attendances [42], therefore for LGA’s with small counts, it is possible the numbers are driven by a small number of at-risk young people. To better understand the service impact across Australia, researchers should utilise linked data to better identify where individuals are seeking help and their access to the health service system.

### Conclusions

The current paper provides a post COVID-19 overview of ambulance attendances for adolescent suicidal and NSSI behaviours across three eastern Australian states, identifying and describing the most disproportionately impacted regions. The high prevalence of attendance across metropolitan regions and high rates per capita in regional and remote settings demonstrate that despite the presence of specialised mental health supports across Australia, more accessible primary and secondary supports are needed to prevent young people from needing to go the ED. These findings also highlight the value of emergency and other real-time data in identifying areas of high demand, guiding service planning, and evaluating current and future models of care. With rates of adolescent mental health at concerningly high levels, it is imperative governments and policy makers invest in accessible primary and secondary mental health services across all regions to improve outcomes for young people and reduce their need to utilise emergency services.

## Supplementary Information

Below is the link to the electronic supplementary material.

Supplementary Material 1

## Data Availability

Due to ethics approvals and data sharing agreements with ambulance services, the data used in this research is not publicly available. .
