# Supplementary material for: Mapping Adolescent Suicidal and Non-Suicidal Self-Injurious Behaviours Across Eastern Australia
Source: J Community Health. 2025 Nov 19;51(2):256–64. doi: 10.1007/s10900-025-01523-0 (PMC13241416; doi:10.1007/s10900-025-01523-0)
Supplement: Supplementary file 1 — Supplementary Material 1 [file 10900_2025_1523_MOESM1_ESM.docx]

#### Table S1. Local government area characteristics and adolescent self-harm attendances, by state (2021–2023)

| Local Government Area | Adolescent Population (2021) | Three Year Average Attendance | Three Year Average Attendance rate per 10,000 | Area km2 | Remoteness | Socioeconomic Status (SES) |
| --- | --- | --- | --- | --- | --- | --- |
|  | New South Wales | | | | | |
| Albury | 4095 | 70.67 | 172.67 | 306 | Inner Regional | Medium SES |
| Armidale Regional | 2468 | 42.00 | 170.33 | 7809 | Inner Regional | Medium SES |
| Ballina | 3224 | 35.33 | 109.67 | 485 | Inner Regional | High SES |
| Balranald | 176 | n.a | n.a | 21691 | Outer Regional | Medium SES |
| Bathurst Regional | 3690 | 72.00 | 195.00 | 3818 | Inner Regional | Medium SES |
| Bayside (NSW) | 9269 | 51.67 | 55.67 | 51 | Major City | High SES |
| Bega Valley | 2436 | 20.67 | 85.00 | 6279 | Outer Regional | Medium SES |
| Bellingen | 1050 | 15.67 | 149.00 | 1600 | Outer Regional | Medium SES |
| Berrigan | 631 | <5.00 | <79.24 | 2066 | Inner Regional | Medium SES |
| Blacktown | 31638 | 341.33 | 108.00 | 239 | Major City | High SES |
| Bland | 410 | 8.67 | 184.00 | 8558 | Outer Regional | Medium SES |
| Blayney | 614 | 6.67 | 69.33 | 1525 | Inner Regional | Medium SES |
| Blue Mountains | 6058 | 73.00 | 96.67 | 1431 | Major City | High SES |
| Bogan | 189 | <5.00 | <264.55 | 14600 | Remote/Very Remote | Medium SES |
| Bourke | 163 | 5.00 | 217.33 | 41598 | Remote/Very Remote | Medium SES |
| Brewarrina | 109 | <5.00 | <458.72 | 19162 | Remote/Very Remote | Low SES |
| Broken Hill | 1194 | 42.67 | 357.33 | 170 | Outer Regional | Low SES |
| Burwood | 2033 | 23.33 | 114.67 | 7 | Major City | High SES |
| Byron | 2438 | 34.00 | 139.33 | 566 | Inner Regional | High SES |
| Cabonne | 1235 | 7.33 | 59.33 | 6022 | Inner Regional | High SES |
| Camden | 9827 | 92.67 | 94.33 | 202 | Major City | High SES |
| Campbelltown (NSW) | 13991 | 215.67 | 154.33 | 311 | Major City | Medium SES |
| Canada Bay | 5247 | 16.00 | 30.67 | 20 | Major City | High SES |
| Canterbury-Bankstown | 27979 | 117.67 | 42.00 | 110 | Major City | Medium SES |
| Carrathool | 186 | <5.00 | <268.82 | 18934 | Remote/Very Remote | Medium SES |
| Central Coast (NSW) | 25841 | 321.67 | 124.33 | 1681 | Major City | Medium SES |
| Central Darling | 102 | n.a | n.a | 53492 | Remote/Very Remote | Low SES |
| Cessnock | 4851 | 65.67 | 135.33 | 1965 | Inner Regional | Low SES |
| Clarence Valley | 3747 | 59.67 | 159.33 | 10429 | Inner Regional | Low SES |
| Cobar | 291 | <5.00 | <171.82 | 45575 | Remote/Very Remote | Medium SES |
| Coffs Harbour | 5895 | 153.67 | 260.67 | 1174 | Inner Regional | Medium SES |
| Coolamon | 374 | <5.00 | <133.69 | 2431 | Inner Regional | Medium SES |
| Coonamble | 278 | 8.67 | 312.00 | 9916 | Remote/Very Remote | Low SES |
| Cootamundra-Gundagai Regional | 867 | 29.67 | 342.00 | 3981 | Inner Regional | Low SES |
| Cowra | 926 | 11.00 | 119.00 | 2809 | Inner Regional | Low SES |
| Cumberland | 15778 | 92.00 | 58.67 | 73 | Major City | Medium SES |
| Dubbo Regional | 4238 | 112.00 | 264.33 | 7535 | Inner Regional | Medium SES |
| Dungog | 722 | 10.00 | 138.67 | 2250 | Inner Regional | Medium SES |
| Edward River | 635 | 8.67 | 136.33 | 8883 | Inner Regional | Medium SES |
| Eurobodalla | 2582 | 31.00 | 120.00 | 3428 | Inner Regional | Medium SES |
| Fairfield | 17019 | 92.00 | 54.00 | 102 | Major City | Low SES |
| Federation | 854 | 5.33 | 62.33 | 5685 | Inner Regional | Low SES |
| Forbes | 871 | 10.33 | 118.33 | 4710 | Outer Regional | Medium SES |
| Georges River | 9636 | 49.00 | 50.67 | 38 | Major City | High SES |
| Gilgandra | 307 | <5.00 | <162.867 | 4832 | Outer Regional | Low SES |
| Glen Innes Severn | 612 | 15.00 | 245.00 | 5480 | Outer Regional | Low SES |
| Goulburn Mulwaree | 2355 | 37.67 | 159.67 | 3220 | Inner Regional | Medium SES |
| Greater Hume Shire | 1045 | 10.33 | 98.67 | 5749 | Inner Regional | Medium SES |
| Griffith | 2103 | 17.33 | 82.67 | 1639 | Outer Regional | Medium SES |
| Gunnedah | 977 | 16.67 | 170.33 | 4987 | Outer Regional | Medium SES |
| Gwydir | 324 | <5.00 | <154.32 | 9260 | Outer Regional | Medium SES |
| Hawkesbury | 5348 | 48.33 | 90.67 | 2775 | Major City | High SES |
| Hay | 180 | 5.33 | 296.67 | 11326 | Outer Regional | Low SES |
| Hilltops | 1458 | 17.00 | 116.33 | 7141 | Inner Regional | Medium SES |
| Hornsby | 12469 | 88.33 | 70.67 | 455 | Major City | High SES |
| Hunters Hill | 1207 | 6.67 | 55.00 | 6 | Major City | High SES |
| Inner West | 9530 | 84.67 | 89.00 | 35 | Major City | High SES |
| Inverell | 1449 | 23.67 | 163.00 | 9405 | Outer Regional | Low SES |
| Junee | 428 | <5.00 | <116.82 | 2030 | Inner Regional | Low SES |
| Kempsey | 2190 | 46.00 | 210.00 | 3376 | Inner Regional | Low SES |
| Kiama | 1624 | 9.67 | 59.33 | 258 | Inner Regional | High SES |
| Ku-ring-gai | 11657 | 85.67 | 73.33 | 85 | Major City | High SES |
| Kyogle | 670 | 10.00 | 149.00 | 3584 | Inner Regional | Low SES |
| Lachlan | 417 | 6.00 | 144.00 | 14968 | Outer Regional | Medium SES |
| Lake Macquarie | 15657 | 300.33 | 191.67 | 649 | Major City | Medium SES |
| Lane Cove | 2573 | 24.67 | 96.00 | 10 | Major City | High SES |
| Leeton | 1254 | 14.00 | 112.00 | 1167 | Outer Regional | Low SES |
| Lismore | 3443 | 87.33 | 253.67 | 1288 | Inner Regional | Medium SES |
| Lithgow | 1479 | 24.00 | 162.33 | 4512 | Inner Regional | Low SES |
| Liverpool | 19944 | 183.00 | 91.67 | 306 | Major City | Medium SES |
| Liverpool Plains | 586 | 7.00 | 119.33 | 5082 | Outer Regional | Low SES |
| Lockhart | 248 | <5.00 | <201.63 | 2896 | Outer Regional | Medium SES |
| Maitland | 7377 | 169.67 | 230.00 | 392 | Major City | Medium SES |
| Mid-Coast | 6422 | 93.67 | 146.00 | 10054 | Inner Regional | Low SES |
| Mid-Western Regional | 1986 | 21.00 | 105.67 | 8752 | Inner Regional | Medium SES |
| Moree Plains | 864 | 7.00 | 81.00 | 17903 | Outer Regional | Medium SES |
| Mosman | 2117 | 9.67 | 45.67 | 9 | Major City | High SES |
| Murray River | 875 | <5.00 | <57.14 | 11863 | Inner Regional | Medium SES |
| Murrumbidgee | 218 | <5.00 | <229.36 | 6881 | Outer Regional | Medium SES |
| Muswellbrook | 1396 | 39.00 | 279.33 | 3405 | Inner Regional | Low SES |
| Nambucca Valley | 1473 | 17.00 | 115.33 | 1491 | Outer Regional | Low SES |
| Narrabri | 982 | 10.33 | 105.33 | 13015 | Outer Regional | Medium SES |
| Narrandera | 446 | 7.67 | 172.00 | 4116 | Outer Regional | Low SES |
| Narromine | 531 | 12.00 | 226.00 | 5262 | Outer Regional | Medium SES |
| Newcastle | 10715 | 229.33 | 214.00 | 187 | Major City | High SES |
| North Sydney | 2645 | 47.67 | 180.33 | 10 | Major City | High SES |
| Northern Beaches | 21146 | 138.67 | 65.67 | 254 | Major City | High SES |
| Oberon | 396 | <5.00 | <126.26 | 3625 | Inner Regional | Medium SES |
| Orange | 3830 | 114.33 | 298.33 | 284 | Inner Regional | Medium SES |
| Parkes | 1185 | 17.33 | 146.33 | 5958 | Outer Regional | Low SES |
| Parramatta | 14938 | 128.33 | 86.00 | 84 | Major City | High SES |
| Penrith | 17013 | 192.00 | 112.67 | 405 | Major City | High SES |
| Port Macquarie-Hastings | 6383 | 87.67 | 137.33 | 3682 | Inner Regional | Medium SES |
| Port Stephens | 5731 | 100.00 | 174.67 | 858 | Inner Regional | Medium SES |
| Queanbeyan-Palerang Regional | 4545 | 72.33 | 159.33 | 5319 | Major City | High SES |
| Randwick | 7809 | 65.67 | 84.00 | 36 | Major City | High SES |
| Richmond Valley | 1836 | 63.67 | 346.67 | 3047 | Inner Regional | Low SES |
| Ryde | 7045 | 59.33 | 84.00 | 40 | Major City | High SES |
| Shellharbour | 6050 | 66.00 | 109.00 | 147 | Major City | Medium SES |
| Shoalhaven | 7253 | 99.00 | 136.33 | 4567 | Inner Regional | Medium SES |
| Singleton | 2049 | 24.67 | 120.33 | 4893 | Inner Regional | Medium SES |
| Snowy Monaro Regional | 2046 | 16.00 | 78.33 | 15165 | Outer Regional | High SES |
| Snowy Valleys | 1117 | 13.00 | 116.33 | 8959 | Inner Regional | Low SES |
| Strathfield | 2452 | 13.00 | 53.00 | 14 | Major City | High SES |
| Sutherland Shire | 17565 | 112.00 | 63.67 | 334 | Major City | High SES |
| Sydney | 4353 | 93.00 | 213.67 | 27 | Major City | High SES |
| Tamworth Regional | 5428 | 134.33 | 247.33 | 9884 | Inner Regional | Medium SES |
| Temora | 458 | <5.00 | <109.17 | 2802 | Outer Regional | Medium SES |
| Tenterfield | 446 | 5.00 | 112.00 | 7324 | Outer Regional | Low SES |
| The Hills Shire | 16816 | 111.67 | 66.33 | 386 | Major City | High SES |
| Tweed | 6836 | 71.00 | 103.67 | 1308 | Major City | Medium SES |
| Upper Hunter Shire | 1195 | 7.00 | 58.67 | 8096 | Inner Regional | Medium SES |
| Upper Lachlan Shire | 556 | <5.00 | <89.93 | 7127 | Inner Regional | High SES |
| Uralla | 516 | 6.33 | 122.67 | 3227 | Outer Regional | Medium SES |
| Wagga Wagga | 5478 | 90.67 | 165.67 | 4824 | Inner Regional | Medium SES |
| Walcha | 198 | <5.00 | <252.53 | 6261 | Outer Regional | Medium SES |
| Walgett | 330 | 10.67 | 323.00 | 22308 | Remote/Very Remote | Low SES |
| Warren | 163 | <5.00 | <306.75 | 10754 | Outer Regional | Medium SES |
| Warrumbungle Shire | 753 | 9.00 | 119.67 | 12372 | Outer Regional | Low SES |
| Waverley | 3920 | 39.00 | 99.33 | 9 | Major City | High SES |
| Weddin | 254 | <5.00 | <196.85 | 3415 | Outer Regional | Medium SES |
| Wentworth | 571 | 7.00 | 122.67 | 26256 | Outer Regional | Medium SES |
| Willoughby | 5728 | 47.33 | 82.67 | 22 | Major City | High SES |
| Wingecarribee | 3962 | 38.67 | 97.33 | 2689 | Inner Regional | High SES |
| Wollondilly | 4693 | 32.33 | 69.00 | 2555 | Inner Regional | High SES |
| Wollongong | 15202 | 146.00 | 96.00 | 684 | Major City | High SES |
| Woollahra | 3305 | 29.33 | 89.00 | 12 | Major City | High SES |
| Yass Valley | 1498 | 7.67 | 51.00 | 3995 | Inner Regional | High SES |
|  | Victoria | | | | | |
| Alpine | 1003 | <5.00 | <49.85 | 4788 | Inner Regional | High SES |
| Ararat | 723 | 5.33 | 74.00 | 4211 | Inner Regional | Medium SES |
| Ballarat | 8898 | 141.67 | 159.33 | 739 | Inner Regional | Medium SES |
| Banyule | 8654 | 69.33 | 80.00 | 63 | Major City | High SES |
| Bass Coast | 2572 | 46.33 | 180.33 | 866 | Inner Regional | Medium SES |
| Baw Baw | 4410 | 32.67 | 74.00 | 4028 | Inner Regional | Medium SES |
| Bayside (Vic.) | 8715 | 31.67 | 36.33 | 37 | Major City | High SES |
| Benalla | 925 | 13.67 | 147.67 | 2353 | Inner Regional | Medium SES |
| Boroondara | 14160 | 51.33 | 36.33 | 60 | Major City | High SES |
| Brimbank | 13302 | 120.67 | 90.67 | 123 | Major City | Medium SES |
| Buloke | 450 | <5.00 | <111.11 | 7998 | Outer Regional | Medium SES |
| Campaspe | 2905 | 29.67 | 102.00 | 4519 | Inner Regional | Medium SES |
| Cardinia | 9361 | 84.67 | 90.33 | 1283 | Major City | High SES |
| Casey | 29690 | 161.00 | 54.00 | 409 | Major City | High SES |
| Central Goldfields | 890 | 9.67 | 108.67 | 1533 | Inner Regional | Low SES |
| Colac Otway | 1569 | <5.00 | <31.87 | 3437 | Inner Regional | Medium SES |
| Corangamite | 1241 | 5.33 | 42.67 | 4407 | Inner Regional | Medium SES |
| Darebin | 8208 | 67.00 | 81.67 | 53 | Major City | High SES |
| East Gippsland | 3090 | 34.00 | 110.00 | 20940 | Outer Regional | Medium SES |
| Frankston | 9907 | 130.33 | 131.33 | 130 | Major City | High SES |
| Gannawarra | 700 | <5.00 | <71.43 | 3738 | Outer Regional | Low SES |
| Glen Eira | 10867 | 70.67 | 65.00 | 39 | Major City | High SES |
| Glenelg | 1352 | 10.67 | 78.67 | 6219 | Outer Regional | Low SES |
| Golden Plains | 2298 | 7.00 | 30.33 | 2703 | Inner Regional | High SES |
| Greater Bendigo | 9026 | 111.00 | 123.00 | 3000 | Inner Regional | Medium SES |
| Greater Dandenong | 10228 | 121.33 | 119.00 | 130 | Major City | Low SES |
| Greater Geelong | 18650 | 154.33 | 82.67 | 1248 | Major City | High SES |
| Greater Shepparton | 5346 | 62.67 | 117.33 | 2422 | Inner Regional | Medium SES |
| Hepburn | 1050 | 12.33 | 117.33 | 1473 | Inner Regional | High SES |
| Hindmarsh | 364 | <5.00 | <137.36 | 7524 | Outer Regional | Low SES |
| Hobsons Bay | 5804 | 76.67 | 132.00 | 64 | Major City | High SES |
| Horsham | 1522 | 23.00 | 151.33 | 4267 | Outer Regional | Medium SES |
| Hume | 19232 | 172.33 | 89.67 | 504 | Major City | Medium SES |
| Indigo | 1370 | <5.00 | <36.50 | 2040 | Inner Regional | High SES |
| Kingston (Vic.) | 10885 | 81.00 | 74.33 | 91 | Major City | High SES |
| Knox | 11180 | 65.33 | 58.67 | 114 | Major City | High SES |
| Latrobe (Vic.) | 5504 | 77.67 | 141.00 | 1426 | Inner Regional | Low SES |
| Loddon | 532 | <5.00 | <93.98 | 6696 | Outer Regional | Low SES |
| Macedon Ranges | 4359 | 32.33 | 74.33 | 1748 | Inner Regional | High SES |
| Manningham | 8873 | 30.00 | 33.67 | 113 | Major City | High SES |
| Mansfield | 969 | 6.00 | 62.00 | 3844 | Outer Regional | High SES |
| Maribyrnong | 4290 | 55.67 | 129.67 | 31 | Major City | High SES |
| Maroondah | 8054 | 112.67 | 139.67 | 61 | Major City | High SES |
| Melbourne | 3292 | 108.00 | 328.00 | 38 | Major City | High SES |
| Melton | 15285 | 140.67 | 92.00 | 528 | Major City | Medium SES |
| Mildura | 4311 | 74.33 | 172.67 | 22082 | Outer Regional | Low SES |
| Mitchell | 3731 | 71.00 | 190.33 | 2862 | Inner Regional | Medium SES |
| Moira | 2178 | 35.33 | 162.33 | 4046 | Inner Regional | Low SES |
| Monash | 12704 | 97.33 | 76.67 | 81 | Major City | High SES |
| Moonee Valley | 7904 | 54.33 | 68.67 | 43 | Major City | High SES |
| Moorabool | 2990 | 30.00 | 100.33 | 2111 | Major City | High SES |
| Moreland | 8806 | 102.33 | 116.33 | 51 | Major City | High SES |
| Mornington Peninsula | 12314 | 78.33 | 63.67 | 724 | Major City | High SES |
| Mount Alexander | 1299 | 10.33 | 79.33 | 1530 | Inner Regional | High SES |
| Moyne | 1438 | 6.33 | 44.33 | 5482 | Inner Regional | High SES |
| Murrindindi | 1049 | 10.67 | 101.67 | 3880 | Inner Regional | Medium SES |
| Nillumbik | 5567 | 59.00 | 105.67 | 432 | Major City | High SES |
| Northern Grampians | 719 | 6.33 | 87.67 | 5730 | Inner Regional | Low SES |
| Port Phillip | 4027 | 44.00 | 109.33 | 21 | Major City | High SES |
| Pyrenees | 505 | <5.00 | <99.01 | 3435 | Inner Regional | Medium SES |
| Queenscliffe | 155 | <5.00 | <322.58 | 9 | Inner Regional | High SES |
| South Gippsland | 2241 | 17.00 | 76.00 | 3296 | Inner Regional | Medium SES |
| Southern Grampians | 1267 | 8.33 | 65.67 | 6654 | Inner Regional | Medium SES |
| Stonnington | 5134 | 30.33 | 59.33 | 26 | Major City | High SES |
| Strathbogie | 697 | 26.67 | 382.33 | 3303 | Inner Regional | Medium SES |
| Surf Coast | 2876 | 17.67 | 61.00 | 1553 | Inner Regional | High SES |
| Swan Hill | 1508 | 15.67 | 104.33 | 6116 | Outer Regional | Medium SES |
| Towong | 402 | <5.00 | <124.38 | 6675 | Outer Regional | Medium SES |
| Unincorporated Vic |  | n.a | n.a | 283 | Outer Regional | High SES |
| Wangaratta | 2289 | 24.67 | 107.67 | 3645 | Inner Regional | Medium SES |
| Warrnambool | 2634 | 30.00 | 114.00 | 121 | Inner Regional | Medium SES |
| Wellington | 3289 | 46.00 | 139.67 | 10817 | Inner Regional | Medium SES |
| West Wimmera | 234 | n.a | n.a | 9109 | Outer Regional | Medium SES |
| Whitehorse | 11891 | 71.00 | 60.00 | 64 | Major City | High SES |
| Whittlesea | 16749 | 96.67 | 57.67 | 490 | Major City | Medium SES |
| Wodonga | 3421 | 39.67 | 116.00 | 433 | Inner Regional | Medium SES |
| Wyndham | 21807 | 150.67 | 69.00 | 542 | Major City | High SES |
| Yarra | 3119 | 30.67 | 98.33 | 20 | Major City | High SES |
| Yarra Ranges | 11761 | 133.33 | 113.33 | 2468 | Major City | High SES |
| Yarriambiack | 492 | <5.00 | <101.63 | 7326 | Outer Regional | Low SES |
|  | Tasmania | | | | | |
| Break O'Day | 299 | <5.00 | <167.22 | 3524 | Outer Regional | Low SES |
| Brighton | 1593 | 18.33 | 115.00 | 171 | Inner Regional | Low SES |
| Burnie | 1618 | 27.33 | 169.00 | 611 | Outer Regional | Low SES |
| Central Coast (Tas.) | 1655 | 19.67 | 119.00 | 933 | Outer Regional | Low SES |
| Central Highlands (Tas.) | 160 | n.a | n.a | 7982 | Outer Regional | Low SES |
| Circular Head | 580 | <5.00 | <86.21 | 4898 | Outer Regional | Low SES |
| Clarence | 4204 | 37.00 | 88.00 | 378 | Inner Regional | High SES |
| Derwent Valley | 883 | 8.33 | 94.67 | 4108 | Inner Regional | Low SES |
| Devonport | 1991 | 73.00 | 366.67 | 111 | Inner Regional | Low SES |
| Dorset | 487 | <5.00 | <102.67 | 3231 | Outer Regional | Low SES |
| Flinders (Tas.) | 34 | n.a | n.a | 1997 | Remote/Very Remote | Medium SES |
| George Town | 512 | <5.00 | <97.66 | 653 | Outer Regional | Low SES |
| Glamorgan-Spring Bay | 190 | <5.00 | <263.16 | 2592 | Remote/Very Remote | Low SES |
| Glenorchy | 3288 | 49.33 | 149.67 | 121 | Inner Regional | Low SES |
| Hobart | 3274 | 41.33 | 126.33 | 78 | Inner Regional | High SES |
| Huon Valley | 1294 | 7.67 | 59.00 | 5507 | Outer Regional | Medium SES |
| Kentish | 488 | <5.00 | <102.46 | 1156 | Outer Regional | Low SES |
| King Island | 73 | n.a | n.a | 1096 | Remote/Very Remote | Medium SES |
| Kingborough | 3118 | 32.00 | 102.67 | 720 | Inner Regional | High SES |
| Latrobe (Tas.) | 842 | 20.00 | 237.33 | 600 | Outer Regional | Medium SES |
| Launceston | 4967 | 48.33 | 97.67 | 1414 | Inner Regional | Low SES |
| Meander Valley | 1484 | 5.00 | 33.67 | 3331 | Inner Regional | Medium SES |
| Northern Midlands | 943 | <5.00 | <53.02 | 5135 | Inner Regional | Medium SES |
| Sorell | 1121 | 14.33 | 128.00 | 584 | Inner Regional | Medium SES |
| Southern Midlands | 497 | <5.00 | <100.60 | 2615 | Outer Regional | Low SES |
| Tasman | 150 | <5.00 | <333.33 | 660 | Outer Regional | Low SES |
| Waratah-Wynyard | 1014 | 11.00 | 108.33 | 3536 | Outer Regional | Low SES |
| West Coast | 219 | <5.00 | <228.31 | 9584 | Remote/Very Remote | Low SES |
| West Tamar | 1882 | 5.67 | 30.33 | 691 | Inner Regional | Medium SES |

Note. To prevent identification of individuals, any average attendance less than 5.00 has been suppressed and reported as "<5.00". Corresponding average attendance rates have been calculated using the maximum possible count of 5. n.a. indicates data could not be reported due to missing population data or no relevant attendances during the study period.
